# Dietary patterns and Subjective cognitive decline in Teacher's Aging Propject (TAP)

**DOI:** 10.1002/alz70860_103274

**Published:** 2025-12-23

**Authors:** Siya Shan, Ting Shen

**Affiliations:** ^1^ Zhejiang University School of Medicine, HangZhou, ZheJiang, China; ^2^ Zhejiang University, HangZhou, ZheJiang, China

## Abstract

**Background:**

Subjective cognitive decline (SCD), characterized by self‐reported cognitive difficulties, may serve as an early indicator of dementia and is linked to an increased risk of mild cognitive impairment (MCI). Diet, as a modifiable risk factor, offers potential for cost‐effective prevention. However, research on dietary patterns and SCD remains limited and inconsistent.

**Method:**

The Teacher's Aging Project (TAP) is a population‐based study initiated in 2022 among schoolteachers in Zhejiang Province, China. Among 3,560 participants with complete data (2022‐2023), several healthy dietary patterns were identified using a simplified food frequency questionnaire (FFQ), including the Chinese Healthy Eating Index (CHEI), Dietary Approaches to Stop Hypertension (DASH), Alternate Mediterranean Diet (aMed), Healthful Plant‐Based Diet Index (hPDI), and MIND. SCD was assessed using seven yes/no questions on perceived recent changes in memory and cognition. Multinomial logistic regression was employed to examine associations between dietary patterns and categorical SCD, with diet scores divided into tertiles. All models were adjusted for covariates, and results were reported as odds ratios (ORs) with 95% confidence intervals (CIs).

**Result:**

The mean age of participants was 49.9 (3.8), with 74% females. Higher adherence to the DASH, AMED, and MIND dietary patterns was significantly linked to a reduced risk of severe SCD, even after adjusting for sociodemographic features, disease history, depression, and anxiety in Model 3 (OR = 0.70, 95% CI: 0.56, 0.89). The AMED diet showed an OR of 0.78 (95% CI: 0.64, 0.95) in Model 1 and a non‐significant trend in Model 3 (OR = 0.85, 95% CI: 0.68, 1.06). The MIND diet demonstrated a significant association with severe SCD (OR = 0.64, 95% CI: 0.52, 0.79 in Model 1 and OR = 0.72, 95% CI: 0.57, 0.90 in Model 3). The effect for moderate SCD was weaker, with most results not statistically significant.

**Conclusion:**

Higher adherence to the DASH, AMED, and MIND dietary patterns is associated with a reduced risk of severe SCD in a middle‐aged population, suggesting that healthier diets may mitigate the risk of cognitive decline.

